# 4-Meth­oxy-2-[(*E*)-(phenyl­imino)meth­yl]phenol

**DOI:** 10.1107/S1600536808026883

**Published:** 2008-08-23

**Authors:** Selma Uçan, Aslı Öztürk, Nursabah Sarıkavaklı, Tuncer Hökelek

**Affiliations:** a Niğde University, Department of Chemistry, 51200, Niğde, Turkey; b Hacettepe University, Department of Physics, 06800 Beytepe, Ankara, Turkey; c Adnan Menderes University, Department of Chemistry, 09010, Aydın, Turkey

## Abstract

In the mol­ecule of the title compound, C_14_H_13_NO_2_, the two aromatic rings are oriented at a dihedral angle of 0.78 (20)°; with the exception of two methyl H atoms the mol­ecule is essentially planar. The intra­molecular O—H⋯N hydrogen bond results in the formation of a non-planar, six-membered ring, which adopts a flattened-boat conformation. In the crystal structure, inter­molecular C—H⋯O hydrogen bonds link the mol­ecules to form parallel networks. There is a C—H⋯π contact between the methyl group and the benzene ring. A π–π contact between the benzene and phenyl rings [centroid–centroid distance = 4.681 (5) Å] is also observed.

## Related literature

For general background, see: Hökelek *et al.* (2004 ▶); Uçan & Mercimek (2005 ▶); Uçan *et al.* (2005 ▶); Garg & Kumar (2003 ▶); Mokles & Elzaher (2001 ▶); Amirnasr *et al.* (2002 ▶); Bella *et al.* (2004 ▶); Chandra & Kumar (2005 ▶); Ray *et al.* (2003 ▶); Yang *et al.* (2000 ▶). For bond-length data, see: Allen *et al.* (1987 ▶). For ring conformation puckering parameters, see: Cremer & Pople (1975 ▶).
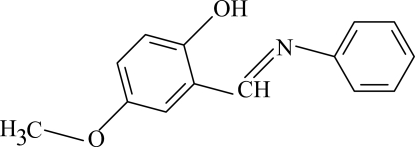


## Experimental

### 

#### Crystal data

C_14_H_13_NO_2_
*M* *_r_* = 227.26
Monoclinic, 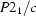

*a* = 20.935 (2) Å
*b* = 4.7151 (10) Å
*c* = 12.275 (3) Å
β = 106.623 (14)°
*V* = 1161.1 (4) Å^3^
*Z* = 4
Mo *K*α radiation
μ = 0.09 mm^−1^
*T* = 294 (2) K
0.40 × 0.20 × 0.10 mm

#### Data collection

Enraf–Nonius TurboCAD-4 diffractometer
Absorption correction: ψ scan (North *et al.*, 1968 ▶) *T*_min_ = 0.971, *T*_max_ = 0.990
1653 measured reflections
1560 independent reflections
521 reflections with *I* > 2σ(*I*)
*R*_int_ = 0.048
θ_max_ = 23.1°
3 standard reflections frequency: 120 min intensity decay: 1%

#### Refinement

*R*[*F* ^2^ > 2σ(*F* ^2^)] = 0.064
*wR*(*F* ^2^) = 0.207
*S* = 0.92
1560 reflections
163 parameters
1 restraint
H atoms treated by a mixture of independent and constrained refinement
Δρ_max_ = 0.20 e Å^−3^
Δρ_min_ = −0.20 e Å^−3^

### 

Data collection: *CAD-4 EXPRESS* (Enraf–Nonius, 1994 ▶); cell refinement: *CAD-4 EXPRESS*; data reduction: *XCAD4* (Harms & Wocadlo, 1995 ▶); program(s) used to solve structure: *SHELXS97* (Sheldrick, 2008 ▶); program(s) used to refine structure: *SHELXL97* (Sheldrick, 2008 ▶); molecular graphics: *ORTEP-3 for Windows* (Farrugia, 1997 ▶) and *PLATON* (Spek, 2003 ▶); software used to prepare material for publication: *WinGX* publication routines (Farrugia, 1999 ▶) and *PLATON*.

## Supplementary Material

Crystal structure: contains datablocks I, global. DOI: 10.1107/S1600536808026883/wn2275sup1.cif

Structure factors: contains datablocks I. DOI: 10.1107/S1600536808026883/wn2275Isup2.hkl

Additional supplementary materials:  crystallographic information; 3D view; checkCIF report

## Figures and Tables

**Table 1:** Hydrogen-bond geometry (Å, °)

*D*—H⋯*A*	*D*—H	H⋯*A*	*D*⋯*A*	*D*—H⋯*A*
O1—H1⋯N1	0.86 (5)	1.82 (5)	2.604 (7)	152 (5)
C7—H7⋯O1^i^	1.03 (5)	2.55 (6)	3.493 (10)	151 (4)
C14—H14*A*⋯O2^ii^	0.96	2.57	3.500 (6)	164
C14—H14*B*⋯*Cg*2^iii^	0.96	3.27	4.142 (8)	152

Symmetry codes: (i) 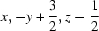; (ii) 
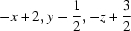
; (iii) 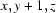. *Cg*2 is the centroid of ring C8–C13.

**Fig. 1.**
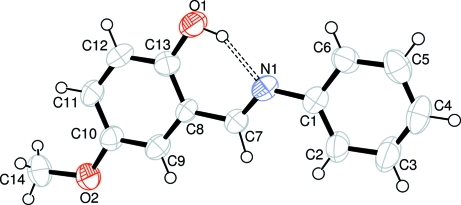
The molecular structure of the title molecule with the atom-numbering scheme. Displacement ellipsoids are drawn at the 50% probability level. The intramolecular hydrogen bond is shown as a double dashed line.

**Fig. 2.**
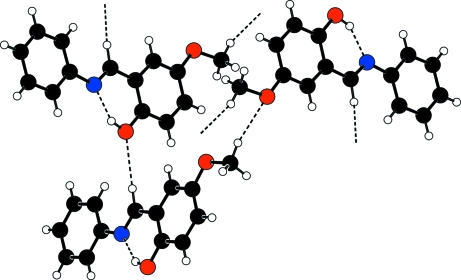
A part of the crystal structure of the title compound, showing the formation of the network structure. Hydrogen bonds are shown as dashed lines.

**Fig. 3.**
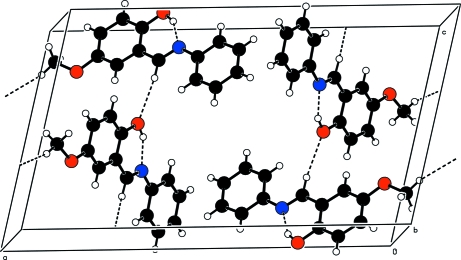
A partial packing diagram of the title compound. Hydrogen bonds are shown as dashed lines.

## supplementary crystallographic information

### Comment

Few classes of coordination compounds have been the subject of as much attention as Schiff base complexes formed by the condensation of amines with carbonyl derivatives (Hökelek *et al.*, 2004; Uçan & Mercimek, 2005; Uçan *et al.*, 2005). Schiff bases of diamines and their complexes have a variety of applications including biological, clinical and analytical (Garg & Kumar, 2003; Mokles & Elzaher, 2001; Amirnasr *et al.*, 2002). A great number of Schiff base complexes with metals have provoked wide interest because they possess a diverse spectrum of biological and pharmaceutical activities, such as antitumor and antioxidative activities, as well as the inhibition of lipid peroxidation (Bella *et al.*, 2004; Chandra & Kumar, 2005; Ray *et al.*, 2003; Yang *et al.*, 2000). We report here the crystal structure of the title compound.

In the molecule of the title compound, (Fig. 1) the bond lengths (Allen *et al.*, 1987) and angles are generally within normal ranges. Rings A (C1—C6) and B (C8—C13) are, of course, planar, and they are oriented at a dihedral angle of 0.78 (20)°; with the exception of two methyl H atoms the molecule is essentially planar. It is known that Schiff bases may exhibit thermochromism or photochromism, depending on the planarity or non-planarity of the molecule, respectively. Therefore, one can expect thermochromic properties in the title compound as a result of the planarity of the molecule. The intramolecular O—H···N hydrogen bond (Table 1) results in the formation of a non-planar, six-membered ring C (N1/O1/C7/C8/C13/H1); this adopts a flattened-boat conformation having a total puckering amplitude, Q_T_, of 0.381 (3) Å (Cremer & Pople, 1975).

In the crystal structure, intermolecular C—H···O hydrogen bonds (Table 1) link the molecules to form a network structure (Fig. 2), in which they are arranged parallel to each other (Fig. 3). A C—H···π contact (Table 1) between the methyl group and B ring is observed. A π—π contact between the A and B rings *Cg*1···*Cg*2^i^ [symmetry code: (i) *x*, *y* - 1, *z*, where *Cg*1 and *Cg*2 are the centroids of the rings A and B, respectively, further stabilizes the structure, with a centroid-centroid distance of 4.681 (5) Å.

### Experimental

The title compound was prepared by the usual condensation method. Aniline (0.931 g, 10 mmol) was dissolved in methanol (10 ml) and added to a solution of 4-methoxysalicylaldehyde (3.042 g, 20 mmol) in methanol (10 ml). The reaction mixture was stirred for 3 h and left overnight at 298 K. The resulting precipitate was filtered and washed with cold ethanol. It was recrystalized from dichloromethane, dried in a vacuum desiccator and the purity was checked by TLC (yield; 3.854 g, 84%, m.p. 341 K).

### Refinement

H1 (attached to O1) and H7 (attached to C7) were located in difference syntheses and refined isotropically [O—H = 0.86 (5) Å and *U*_iso_(H) = 0.05 (3) Å^2^; C—H = 1.03 (5) Å and *U*_iso_(H) = 0.039 (18) Å^2^]. The remaining H atoms were positioned geometrically, with C—H = 0.93 and 0.96 Å for aromatic and methyl H, respectively, and constrained to ride on their parent atoms with *U*_iso_(H) = *xU*_eq_(C), where *x* = 1.5 for methyl H and *x* = 1.2 for aromatic H atoms. A restraint on the O—H bond was applied.

### Crystal data

**Table:**

C_14_H_13_NO_2_	*F*_000_ = 480
*M**_r_* = 227.26	*D*_x_ = 1.300 Mg m^−^^3^
Monoclinic, *P*2_1_/*c*	Mo *K*α radiation λ = 0.71073 Å
Hall symbol: -P 2ybc	Cell parameters from 25 reflections
*a* = 20.935 (2) Å	θ = 5.2–17.4º
*b* = 4.7151 (10) Å	µ = 0.09 mm^−^^1^
*c* = 12.276 (3) Å	*T* = 294 (2) K
β = 106.623 (14)º	Rod-shaped, orange
*V* = 1161.1 (4) Å^3^	0.40 × 0.20 × 0.10 mm
*Z* = 4	

### Data collection

**Table:**

Enraf–Nonius TurboCAD-4 diffractometer	*R*_int_ = 0.048
Radiation source: fine-focus sealed tube	θ_max_ = 23.1º
Monochromator: graphite	θ_min_ = 3.3º
*T* = 294(2) K	*h* = −22→21
non–profiled ω scans	*k* = 0→5
Absorption correction: ψ scan(North *et al.*, 1968)	*l* = 0→13
*T*_min_ = 0.971, *T*_max_ = 0.990	3 standard reflections
1653 measured reflections	every 120 min
1560 independent reflections	intensity decay: 1%
521 reflections with *I* > 2σ(*I*)	

### Refinement

**Table:**

Refinement on *F*^2^	Secondary atom site location: difference Fourier map
Least-squares matrix: full	Hydrogen site location: inferred from neighbouring sites
*R*[*F*^2^ > 2σ(*F*^2^)] = 0.064	H atoms treated by a mixture of independent and constrained refinement
*wR*(*F*^2^) = 0.207	*w* = 1/[σ^2^(*F*_o_^2^) + (0.0803*P*)^2^] where *P* = (*F*_o_^2^ + 2*F*_c_^2^)/3
*S* = 0.92	(Δ/σ)_max_ < 0.001
1560 reflections	Δρ_max_ = 0.20 e Å^−^^3^
163 parameters	Δρ_min_ = −0.20 e Å^−^^3^
1 restraint	Extinction correction: none
Primary atom site location: structure-invariant direct methods	

### Special details

**Table:**

Geometry. All e.s.d.'s (except the e.s.d. in the dihedral angle between two l.s. planes) are estimated using the full covariance matrix. The cell e.s.d.'s are taken into account individually in the estimation of e.s.d.'s in distances, angles and torsion angles; correlations between e.s.d.'s in cell parameters are only used when they are defined by crystal symmetry. An approximate (isotropic) treatment of cell e.s.d.'s is used for estimating e.s.d.'s involving l.s. planes.
Refinement. Refinement of *F*^2^ against ALL reflections. The weighted *R*-factor *wR* and goodness of fit *S* are based on *F*^2^, conventional *R*-factors *R* are based on *F*, with *F* set to zero for negative *F*^2^. The threshold expression of *F*^2^ > σ(*F*^2^) is used only for calculating *R*-factors(gt) *etc*. and is not relevant to the choice of reflections for refinement. *R*-factors based on *F*^2^ are statistically about twice as large as those based on *F*, and *R*- factors based on ALL data will be even larger.

### Fractional atomic coordinates and isotropic or equivalent isotropic displacement parameters (Å^2^)

**Table:**

	*x*	*y*	*z*	*U*_iso_*/*U*_eq_	
O1	0.7505 (3)	0.7726 (12)	1.0014 (4)	0.0656 (17)	
H1	0.725 (3)	0.879 (11)	0.951 (4)	0.05 (3)*	
O2	0.9196 (2)	0.2785 (11)	0.7864 (4)	0.0649 (16)	
N1	0.7019 (3)	1.0588 (12)	0.8146 (5)	0.0452 (17)	
C1	0.6566 (3)	1.2627 (15)	0.7496 (6)	0.043 (2)	
C2	0.6507 (4)	1.3416 (17)	0.6390 (7)	0.065 (3)	
H2	0.6792	1.2622	0.6015	0.078*	
C3	0.6041 (4)	1.5335 (18)	0.5837 (7)	0.079 (3)	
H3	0.6003	1.5789	0.5083	0.095*	
C4	0.5626 (4)	1.6608 (17)	0.6377 (8)	0.066 (3)	
H4	0.5314	1.7939	0.5997	0.079*	
C5	0.5677 (4)	1.5897 (17)	0.7486 (8)	0.064 (2)	
H5	0.5399	1.6744	0.7860	0.076*	
C6	0.6144 (4)	1.3919 (15)	0.8039 (6)	0.055 (2)	
H6	0.6177	1.3443	0.8789	0.066*	
C7	0.7415 (4)	0.9240 (16)	0.7710 (7)	0.043 (2)	
H7	0.742 (2)	0.943 (11)	0.688 (5)	0.039 (18)*	
C8	0.7886 (3)	0.7208 (15)	0.8350 (6)	0.0397 (19)	
C9	0.8325 (3)	0.5868 (15)	0.7860 (6)	0.049 (2)	
H9	0.8306	0.6280	0.7111	0.059*	
C10	0.8787 (4)	0.3956 (16)	0.8450 (7)	0.048 (2)	
C11	0.8823 (4)	0.3314 (16)	0.9555 (7)	0.057 (2)	
H11	0.9139	0.2028	0.9962	0.068*	
C12	0.8383 (4)	0.4607 (17)	1.0059 (6)	0.056 (2)	
H12	0.8402	0.4159	1.0805	0.067*	
C13	0.7918 (4)	0.6544 (17)	0.9470 (7)	0.049 (2)	
C14	0.9735 (3)	0.1055 (17)	0.8484 (7)	0.077 (3)	
H14A	0.9978	0.0375	0.7982	0.115*	
H14B	0.9563	−0.0527	0.8805	0.115*	
H14C	1.0026	0.2150	0.9082	0.115*	

### Atomic displacement parameters (Å^2^)

**Table:**

	*U*^11^	*U*^22^	*U*^33^	*U*^12^	*U*^13^	*U*^23^
O1	0.085 (4)	0.070 (4)	0.045 (4)	0.028 (4)	0.023 (3)	0.010 (4)
O2	0.062 (4)	0.072 (4)	0.064 (4)	0.026 (3)	0.025 (3)	0.010 (3)
N1	0.058 (4)	0.033 (4)	0.043 (4)	−0.003 (3)	0.012 (4)	0.001 (3)
C1	0.047 (5)	0.032 (5)	0.046 (5)	−0.010 (4)	0.005 (4)	0.000 (5)
C2	0.067 (6)	0.069 (7)	0.055 (6)	0.028 (5)	0.011 (5)	0.004 (5)
C3	0.089 (7)	0.074 (7)	0.063 (6)	0.034 (6)	0.005 (6)	0.015 (6)
C4	0.071 (7)	0.038 (6)	0.072 (7)	0.007 (5)	−0.008 (5)	0.003 (5)
C5	0.052 (6)	0.048 (6)	0.092 (8)	0.001 (5)	0.023 (5)	−0.006 (5)
C6	0.062 (5)	0.043 (5)	0.068 (6)	0.006 (5)	0.031 (5)	0.006 (5)
C7	0.046 (5)	0.041 (5)	0.040 (5)	−0.003 (4)	0.008 (4)	−0.002 (5)
C8	0.046 (5)	0.033 (5)	0.036 (5)	0.003 (4)	0.007 (4)	0.003 (4)
C9	0.059 (5)	0.043 (5)	0.040 (5)	−0.002 (5)	0.008 (4)	−0.001 (4)
C10	0.052 (5)	0.037 (5)	0.053 (6)	0.001 (4)	0.014 (4)	0.010 (5)
C11	0.058 (5)	0.052 (6)	0.058 (6)	0.013 (5)	0.014 (5)	0.022 (5)
C12	0.068 (6)	0.062 (6)	0.034 (5)	0.000 (5)	0.010 (4)	0.006 (5)
C13	0.054 (5)	0.048 (6)	0.046 (5)	−0.002 (4)	0.014 (4)	−0.005 (5)
C14	0.064 (6)	0.074 (6)	0.093 (7)	0.034 (5)	0.025 (5)	0.011 (6)

### Geometric parameters (Å, °)

**Table:**

O1—C13	1.355 (8)	C6—H6	0.9300
O1—H1	0.86 (5)	C7—C8	1.437 (9)
O2—C10	1.381 (8)	C7—H7	1.03 (5)
O2—C14	1.423 (7)	C8—C13	1.393 (8)
N1—C1	1.424 (8)	C9—C8	1.387 (9)
N1—C7	1.277 (8)	C9—C10	1.368 (8)
C1—C2	1.379 (9)	C9—H9	0.9300
C2—C3	1.362 (9)	C11—C10	1.370 (8)
C2—H2	0.9300	C11—C12	1.390 (9)
C3—H3	0.9300	C11—H11	0.9300
C4—C3	1.372 (10)	C12—C13	1.378 (9)
C4—C5	1.376 (9)	C12—H12	0.9300
C4—H4	0.9300	C14—H14A	0.9600
C5—H5	0.9300	C14—H14B	0.9600
C6—C1	1.390 (9)	C14—H14C	0.9600
C6—C5	1.381 (9)		
			
C13—O1—H1	104 (5)	C9—C8—C13	118.3 (7)
C10—O2—C14	117.7 (6)	C9—C8—C7	120.2 (7)
C7—N1—C1	120.6 (7)	C13—C8—C7	121.5 (7)
C2—C1—C6	117.7 (7)	C10—C9—C8	121.8 (7)
C2—C1—N1	126.2 (7)	C10—C9—H9	119.1
C6—C1—N1	116.1 (7)	C8—C9—H9	119.1
C3—C2—C1	121.3 (8)	C9—C10—C11	120.1 (8)
C3—C2—H2	119.3	C9—C10—O2	115.9 (7)
C1—C2—H2	119.3	C11—C10—O2	124.0 (7)
C2—C3—C4	120.8 (9)	C10—C11—C12	119.2 (7)
C2—C3—H3	119.6	C10—C11—H11	120.4
C4—C3—H3	119.6	C12—C11—H11	120.4
C3—C4—C5	119.4 (9)	C13—C12—C11	121.0 (8)
C3—C4—H4	120.3	C13—C12—H12	119.5
C5—C4—H4	120.3	C11—C12—H12	119.5
C4—C5—C6	119.7 (8)	O1—C13—C12	117.9 (8)
C4—C5—H5	120.2	O1—C13—C8	122.5 (7)
C6—C5—H5	120.2	C12—C13—C8	119.6 (8)
C5—C6—C1	121.2 (8)	O2—C14—H14A	109.5
C5—C6—H6	119.4	O2—C14—H14B	109.5
C1—C6—H6	119.4	H14A—C14—H14B	109.5
N1—C7—C8	121.9 (8)	O2—C14—H14C	109.5
N1—C7—H7	125 (3)	H14A—C14—H14C	109.5
C8—C7—H7	113 (3)	H14B—C14—H14C	109.5
			
C7—N1—C1—C2	−1.5 (10)	N1—C7—C8—C13	2.1 (10)
C7—N1—C1—C6	178.3 (7)	C9—C8—C13—O1	−179.3 (7)
C1—N1—C7—C8	178.8 (6)	C7—C8—C13—O1	1.1 (11)
C14—O2—C10—C9	172.5 (6)	C9—C8—C13—C12	0.4 (10)
C14—O2—C10—C11	−7.3 (10)	C7—C8—C13—C12	−179.2 (7)
N1—C1—C2—C3	178.0 (7)	C10—C9—C8—C13	−0.6 (10)
C6—C1—C2—C3	−1.8 (11)	C10—C9—C8—C7	179.0 (6)
C1—C2—C3—C4	1.9 (12)	C8—C9—C10—C11	0.1 (11)
C5—C4—C3—C2	−1.0 (12)	C8—C9—C10—O2	−179.7 (6)
C3—C4—C5—C6	0.1 (12)	C12—C11—C10—C9	0.6 (11)
C5—C6—C1—C2	0.8 (10)	C12—C11—C10—O2	−179.6 (7)
C5—C6—C1—N1	−179.0 (6)	C10—C11—C12—C13	−0.8 (11)
C1—C6—C5—C4	0.0 (11)	C11—C12—C13—O1	−179.9 (7)
N1—C7—C8—C9	−177.5 (7)	C11—C12—C13—C8	0.4 (11)

### Hydrogen-bond geometry (Å, °)

**Table:**

*D*—H···*A*	*D*—H	H···*A*	*D*···*A*	*D*—H···*A*
O1—H1···N1	0.86 (5)	1.82 (5)	2.604 (7)	152 (5)
C7—H7···O1^i^	1.03 (5)	2.55 (6)	3.493 (10)	151 (4)
C14—H14A···O2^ii^	0.96	2.57	3.500 (6)	164
C14—H14B···Cg2^iii^	0.96	3.27	4.142 (8)	152

Symmetry codes: (i) *x*, −*y*+3/2, *z*−1/2; (ii) −*x*+2, *y*−1/2, −*z*+3/2; (iii) *x*, *y*+1, *z*.
